# Successful Hybrid Approach Treatment of a Large Persistent Sciatic Artery Aneurysm—A Case Report

**DOI:** 10.3390/medicina59071328

**Published:** 2023-07-19

**Authors:** Vladimir Cvetic, Marko Miletic, Borivoje Lukic, Dragoslav Nestorovic, Ognjen Kostic, Milos Sladojevic, Petar Zlatanovic, Nenad Jakovljevic

**Affiliations:** 1 Center for Radiology, University Clinical Center of Serbia, Pasterova No. 2, 11000 Belgrade, Serbia; drvladimircvetic@gmail.com (V.C.); boral83@gmail.com (B.L.);; 2 Department of Radiology, Faculty of Medicine, University of Belgrade, Dr Subotica No. 8, 11000 Belgrade, Serbia; 3 Clinic for Vascular and Endovascular Surgery, University Clinical Center of Serbia, Koste Todorovica 8, 11000 Belgrade, Serbia; kosticognjen@ymail.com (O.K.); milos.sladojevic@gmail.com (M.S.); petar91goldy@gmail.com (P.Z.); nesajleo@gmail.com (N.J.); 4 Department of Surgery, Faculty of Medicine, University of Belgrade, Dr Subotica No. 8, 11000 Belgrade, Serbia

**Keywords:** persistent sciatic artery, aneurysm, endovascular embolization, hybrid approach

## Abstract

*Background and Objectives*: Persistent sciatic artery (PSA) is a rare congenital vascular anomaly that is often asymptomatic, but can be associated with aneurysm formation and potential complications, such as thromboembolism or aneurysm rupture in some cases. We present a case of a 75-year-old woman with a symptomatic thrombus-containing aneurysm of the left PSA. *Materials and Methods*: The treatment of the PSA aneurysm involved a successful hybrid approach, which included open surgical bypass and endovascular embolization. The open surgical bypass was performed from the left common femoral artery to the left above-the-knee popliteal artery using a synthetic graft, while the aneurysm exclusion was achieved through endovascular plug embolization. *Results*: Control angiography revealed complete exclusion of the PSA aneurysm. At the 1-month follow-up, there were no palpable pulsatile masses in the left gluteal region, and the patient reported no symptoms. *Conclusions*: Given the high incidence of limb- and life-threatening complications associated with a PSA aneurysm, accurate diagnosis and appropriate treatment are crucial. In this case, a combination of open surgical and endovascular techniques resulted in a favorable outcome for the patient, highlighting the effectiveness of the hybrid approach in managing PSA aneurysms. Further studies are warranted to explore and refine treatment strategies for these complex vascular anomalies.

## 1. Introduction

Persistent sciatic artery (PSA) is a rare embryological vascular anomaly that occurs in a small percentage of the population, ranging from 0.025% to 0.04% [1]. PSA was first reported in 1832, but its angiographical presentation was not described until 1960 [2,3].

During embryological development, the sciatic artery is a continuation of the internal iliac artery and serves as the primary source of blood supply in the first trimester [4]. After the first trimester, the sciatic artery typically regresses, while the femoral artery becomes the main source of blood supply to the lower extremities [5]. In cases where the sciatic artery fails to regress completely, a persistent sciatic artery may develop. Unilateral PSA is more prevalent than bilateral formation and appears on the right side more frequently [6]. 

Most of the patients with a PSA do not experience any symptoms. However, the PSA is associated with the development of aneurysms in 15–44% of the cases, which can lead to further complications, such as thromboembolism, rupture or sciatic nerve compression, resulting in pain [7]. 

## 2. Case Report

A 75-year-old woman with no comorbidities was referred to the Clinic for Vascular and Endovascular Surgery, University Clinical Center of Serbia, for assessment of pain and a pulsatile tumefaction in the left gluteal region. Upon examination, bilateral femoral, popliteal and pedal pulses were palpable. The patient exhibited normal sensation and motor function of the lower extremities. Considering the size and pulsation of the mass, we decided to proceed with a computed tomographic arteriogram (CTA) as the initial diagnostic approach. 

**Figure 1:**
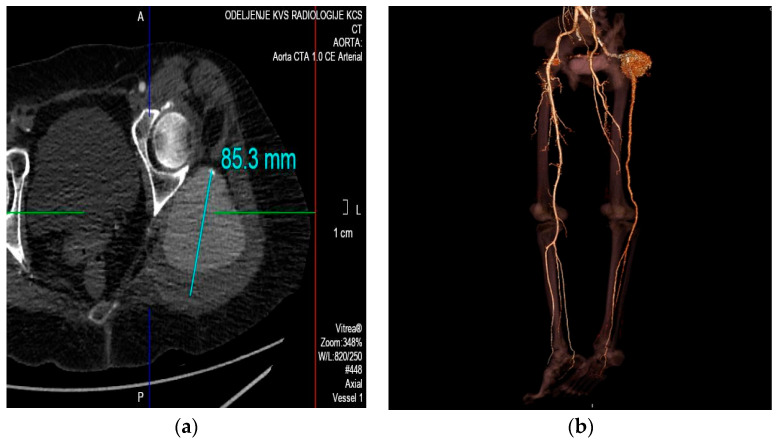
Computed tomographic arteriogram. (**a**) Axial cross section showed a pseudoaneurysm that measured 85 mm in diameter, with the parietal thrombus on posterior wall. (**b**) VR reconstruction revealed a large pseudoaneurysm of the left persistent sciatic artery, as well as the usual anatomical presentation of the right iliac and femoral arteries.

The CTA provided additional information regarding the patient’s condition. It revealed a unilateral left-sided PSA aneurysm measuring 8.5 cm in diameter, with an approximately 2 cm thrombus along the posterior wall, while the arterial lumen remained patent (Figure 1a). Furthermore, the CTA demonstrated normal morphology of the iliac artery, common femoral artery (CFA) and deep femoral artery (DFA). However, the superficial femoral artery (SFA) appeared hypoplastic and lacked the usual anatomical continuation as the popliteal artery (PA). Instead, the PA originated directly from the PSA. Arteries of the right lower limb exhibited normal anatomical characteristics. (Figure 1b).

While the left common femoral artery was patent, the left SFA was hypoplastic and failed to establish a connection with the popliteal artery. Consequently, the PSA assumed a dominant role as the main blood supplier to the left lower limb. As a result, a medical consilium, consisting of an interventional radiologist, vascular surgeon and diagnostic radiologist, proposed an initial treatment plan, consisting of three procedures—open surgical bypass, endovascular embolization of the PSA aneurysm and excision of the aneurysm.

The initial step of the treatment involved an open surgical bypass procedure to redirect the blood flow away from the circulation of the PSA. Following the standard exposure of the CFA, an above-the-knee femoropopliteal bypass was created using an 8 mm polytetrafluoroethylene graft (PTFE—JOTEC GmbH, Hechingen, Germany) as the conduit.

**Figure 2:**
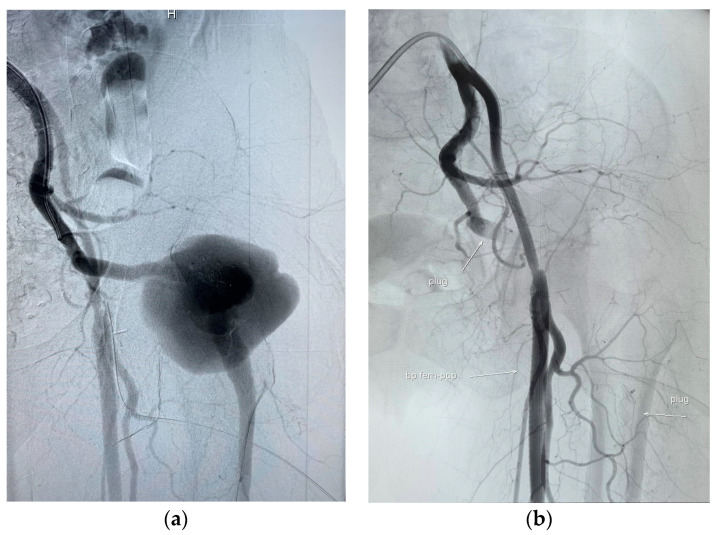
(**a**) Selective digital subtraction angiography (DSA) of the left persistent sciatic artery, confirming a large pseudoaneurysm (H—head); (**b**) DSA after the utilization of sandwich embolization technique. Both inflow and outflow of the pseudoaneurysm were embolized using vascular plugs. Flow through the pseudoaneurysm is not observed after embolization.

Following the successful open surgical bypass, we proceeded with the next step of the treatment, which involved endovascular exclusion of the PSA aneurysm. We opted for a right, retrograde, femoral percutaneous access, considering it to be the most suitable approach. After the placement of a 6 French crossover sheath (Terumo Medical Corp., Elkton Blvd., Elkton, USA), angiography was performed to confirm the presence of the large left-sided PSA aneurysm and the hypoplastic left SFA (Figure 2a). Upon reaching the aneurysm, two Amplatzer Vascular Plugs (AVP—Abbott Medical, North Plymouth, MN, USA) were carefully delivered. The sandwich technique was employed, where a 12 mm AVP was positioned in the outflow vessel of the aneurysm, while a 14 mm plug was placed in the inflow vessel. Subsequent control angiography revealed a complete occlusion of the PSA aneurysm (Figure 2b). 

The procedure was successfully completed without encountering any complications. The patient’s postoperative recovery was uneventful, and she was discharged from the hospital after two days, with antiplatelet therapy. At the 1-month follow-up, no pulsatile mass was detected upon examination. Furthermore, the patient reported improved pain symptoms, and she no longer required the use of pain medication.

## 3. Discussion

**Figure 3:**
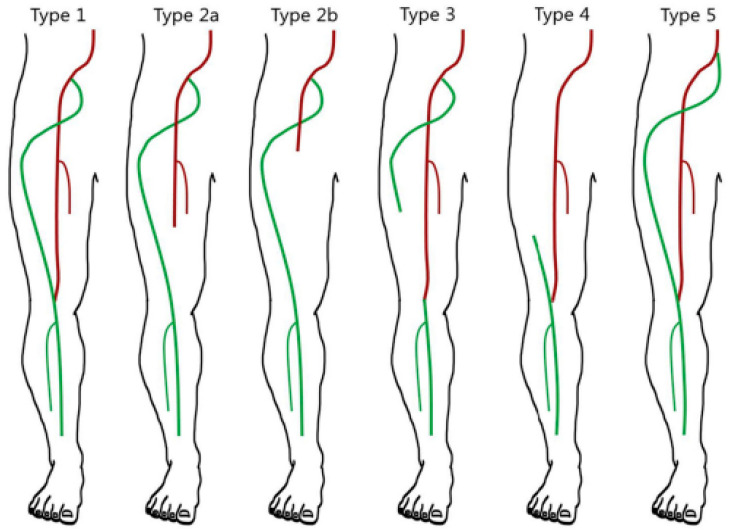
Persistent sciatic artery (PSA) classification by Pillet (1980), modified by Gauffre (1994) [12].

Depending on the development of the SFA, PSA can manifest in either a complete or incomplete form. The complete form of PSA is more commonly observed, accounting for 60–75% of cases, and it involves a hypoplastic SFA and a dominant PSA that serves as the primary blood vessel of the lower [8]. On the other hand, the incomplete form is less prevalent, occurring in 25% of cases, and it involves a dominant SFA as the main inflow vessel of the leg, along with a hypoplastic sciatic artery [9]. The Pillet classification, later modified by Gauffre, is the most widely utilized classification system for PSAs [10,11]. This classification categorizes PSAs into five types, based on the development of both the SFA and PSA (Figure 3). In 2016, Ahn et al. further expanded the previous classification for persistent sciatic arteries by incorporating the presence of an aneurysm, in addition to considering the development of the PSA and SFA [12]. (Table 1).

Our patient presented with a Type 2a, according to the Pillet–Gauffre classification, which corresponds with Class IIIa in the Ahn–Mihn classification. Type 2 PSA represents a complete type of sciatic artery that can be further divided into two subtypes, Type 2a and Type 2b, based on the presence and development of the SFA.

The treatment of symptomatic PSA aneurysms is crucial to prevent potential life-threatening complications, such as rupture and thromboembolism. The choice of treatment approach largely depends on the symptoms exhibited by the patient and the specific form of the PSA [12]. Complete forms of the PSA typically require previous surgical revascularization of the lower limb, which may involve different bypass methods. On the other hand, aneurysms associated with incomplete forms of the PSA can be treated exclusively using endovascular approaches, which may involve utilization of covered stents, endografts or different embolization techniques [13]. Exclusion of a PSA aneurysm can be achieved through either open aneurysmorrhaphy or endovascular interventions, commonly involving endovascular embolization or covered stent implantation [14,15]. 

Taking into account the patient’s age and lifestyle factors, as well as the size of the PSA aneurysm, the treatment plan posed challenges. Solely relying on an endovascular embolization procedure was not feasible due to the dominant nature of the PSA. Exclusion of the PSA through the placement of plugs or coils could compromise the circulation of the leg, resulting in acute ischemia. Additionally, open surgery and direct excision of the aneurysm were ruled out due to the high risk of severe complications. 

In the existing scientific literature, there is a notable absence of documented cases featuring a persistent sciatic artery aneurysm of such significant dimensions. This unique clinical scenario has posed a challenge in determining the most optimal treatment strategy for our patient. The limited available clinical data necessitated a comprehensive evaluation encompassing various factors, including the aneurysm’s size, location and associated symptoms and potential complications of the selected treatment. A thorough assessment of the patient’s medical history, imaging studies and diagnostic findings has been indispensable in gaining a comprehensive understanding of this atypical presentation.

Given the exceptional nature of this case, a multidisciplinary approach involving vascular surgeons, interventional radiologists and diagnostic radiologists has proven essential in making well-informed and effective treatment decisions. Furthermore, a meticulous analysis of the potential risks and benefits associated with different treatment modalities has been crucial. By employing this approach, we aimed to maximize the patient’s outcomes, while minimizing adverse effects and complications.

In a systematic review conducted by Charisis et al., the focus was on the endovascular treatment of persistent sciatic artery aneurysms utilizing primary stenting. The review encompassed 15 case reports, with a median age of 66 years among the patients involved. Following the treatment, all of the patients were asymptomatic, with no recurrence of symptoms. The findings of the study strongly indicated that endovascular treatment with primary stenting for persistent sciatic artery aneurysms is a safe and effective approach in well-selected cases [15].

Based on the precise pseudoaneurysm’s location, the biomechanical forces exerted in the surrounding area, a comprehensive assessment of the aneurysm’s dimensions, the potential occurrence of endoleak and careful consideration of the patient’s sedentary lifestyle factors, we have determined that a solely endovascular intervention through the utilization of a covered stent placement is not deemed a suitable course for the present case. Alternatively, endovascular embolization can be accomplished by coiling or plugs implantation. Since the PSA is a rare embryological vascular anomaly, there are no specific studies comparing the efficacy of plugs and coiling in the treatment of PSA aneurysms. Ryer et al. conducted a comparative study on the efficacy of plugs versus coil embolization in internal iliac artery aneurysm embolization, revealing similar results [16]. In a meta-analysis study by Wong et al., which included 181 patients, the outcomes and cost-effectiveness of internal iliac artery embolization using vascular plugs versus coiling were compared. They demonstrated that vascular plugs were superior to coiling in terms of shorter intervention and fluoroscopy times, resulting in reduced radiation exposure, as well as the total cost of the intervention [17].

The literature reports several cases of PSA aneurysms that have been successfully treated using a hybrid approach [18,19]. It is worth noting that several studies have been conducted on persistent sciatic artery aneurysms, many of which presented with a complete form of PSA akin to our patient. However, it is crucial to acknowledge that the aneurysms addressed in those studies exhibited considerably smaller diameters when compared to the aneurysm observed in our case. 

Acknowledging all the available information, we made the decision to proceed with a hybrid approach, combining open surgical blood flow restoration and endovascular aneurysm exclusion. Initially, we also considered open aneurysmorrhaphy as a backup option, in case endovascular embolization proved insufficient. However, subsequent control diagnostics confirmed the complete exclusion of the PSA pseudoaneurysm, eliminating the need for open exclusion.

## 4. Conclusions

The fast and accurate diagnosis and appropriate treatment of the PSA and its associated complications are crucial due to their potential life-threatening nature. In our case, we successfully employed a hybrid approach combining open revascularization and endovascular embolization to treat a large PSA aneurysm. This comprehensive strategy effectively restored blood flow of the lower limb and excluded the aneurysm, mitigating the risk of complications. The hybrid approach optimizes treatment outcomes by utilizing both surgical and minimally invasive techniques, offering a promising treatment option, particularly in cases of large PSA aneurysms, where surgical extirpation of the aneurysm carries a high risk of complications, such as severe bleeding. Further research is needed to establish standardized protocols and enhance patient outcomes in the management of the PSA and its associated aneurysms.

## Figures and Tables

**Table 1:** Ahn–Mihn classification of PSAs [12].

Class	SFA (Superficial Femoral Artery)	PSA (Persistent Sciatic Artery)	Aneurysm	Pillet–Gauffre Classification
Class I Class Ia	Complete	Complete	− +	Type 1, 5a
Class II Class IIa	Complete	Incomplete	− +	Type 3, 4
Class III Class IIIa	Incomplete	Complete	− +	Type 2a, 2b, 5b
Class IV Class IVa	Incomplete	Incomplete	− +	None
